# Combining photo-elicitation and discourse analysis to examine adolescents’ sexuality in rural Zambia

**DOI:** 10.1186/s12939-022-01662-z

**Published:** 2022-05-03

**Authors:** Chama Mulubwa, Anna-Karin Hurtig, Joseph Mumba Zulu, Charles Michelo, Ingvild Fossgard Sandøy, Isabel Goicolea

**Affiliations:** 1 School of Public Health, University of Zambia, P.O. Box 50110, Lusaka, Zambia; 2 Department of Epidemiology and Global Health, Umeå University, Umeå, SE Sweden; 3 Centre of Infectious Diseases and Research in Zambia, P.O Box 34620, Lusaka, Zambia; 4 Department of Global Public Health and Primary Care, Centre for Intervention Science in Maternal and Child Health (CISMAC), Centre for International Health (CIH), University of Bergen, Bergen, Norway

**Keywords:** Adolescents, Sexuality, Sexual scripts, Interpretative repertoires, Zambia

## Abstract

**Introduction:**

This article aimed to analyse constructions of adolescents’ sexualities and sexual health and the consequences of these discourses for adolescents’ exercise of their sexual reproductive health and rights (SRHR) in rural Zambia.

**Methods:**

Interpretative repertoires, which is rooted in discursive psychology was used to analyse data from photo-elicitations interviews and focus group discussions. Our participants included 25 adolescents who participated in a SRHR intervention that aimed to reduce adolescents’ pregnancies and early marriages.

**Results:**

We identified three interpretative repertories: 1) sex is for mature people in which adolescents positioned themselves as ‘immature, and young to engage in sex; 2) gendered respectful behaviours in which what was considered disrespectful (and respectful) behaviour in relation to sexuality were strongly influenced by gender, and more clearly defined for girls than it was for boys. Sexuality was not only about individual choices but about being respectful to parents; and 3) acquiring and using knowledge about sexuality in which adolescents conflicted between having and applying SRHR knowledge.

**Conclusion:**

These repertories offer an important context that shape how adolescents negotiate, adopt and resist SRHR interventions. Future interventions that target adolescents’ SRHR must aim to address the sexual scripts that serve to erect barriers against positive sexual behaviours, including access to SRHR services that promote safer sex.

## Introduction

Achievement of sexual and reproductive health and rights (SRHR) have far-reaching benefits for adolescents to live healthy and fulfilling lives. Yet, adolescent SRHR remains a neglected issue, especially in sub-Saharan Africa where more than half of the population are young people below the age of 25 years [1, 2]. For example, data across the globe shows that adolescents’ unmet needs for contraception are high, and maternal mortality remains a leading cause of death among adolescents aged 15–19 years [1].

In order to address adolescents SRHR challenges, SRHR interventions targeting adolescents in sub-Saharan Africa have flourished over the last two decades [2]. Although some of these interventions yield positive results, other investments have resulted in no effects or have been counterproductive [1]. We argue that, in order to achieve comprehensive SRHR for adolescents, there is a need for a holistic approach that recognizes the immediate, distal, social and cultural factors shaping adolescents’ sexuality and sexual practices. Therefore, we agree with Hindin’s argument that the success of adolescent SRHR interventions does not only depend on ensuring sustainable resources but must also consider how cultural and social expressions of youth sexuality shape its adoption and adapt their design accordingly [3].

Previous studies have shown that cultural and social norms play a major role in adolescents’ understandings and practices in relation to sexuality and reproduction [4–6]. In many countries, such understandings and practices are not only shaped by laws that often limit adolescents’ autonomy in relation to SRHR, but also by cultural norms, religion, gendered expectations and inequalities. In sub-Saharan Africa, as elsewhere, a complex set of interactions between contextual factors (i.e., poverty, a high disease burden and weak healthcare systems) and political factors (i.e., lack of political will and power) shape how adolescents understand and experience sexuality and SRHR programs [1].

Interventions targeting adolescents’ SRHR have focused on ensuring that essential information and services are available, accessible, acceptable and of good quality [2, 7]. While such aspects are unquestionably relevant, many of these interventions have favoured individualistic approaches, disregarding how social and cultural norms around sexuality shape how adolescents negotiate, adopt and/or resist SRHR interventions. There remains a critical gap in understanding how such norms around sexuality influence the negotiation and adoption of SRHR interventions among adolescents. To fill this gap, this study aimed to analyse discourses on adolescents’ sexuality and sexual health and the consequences of these discourses for adolescents’ exercising of their sexual and reproductive rights in rural Zambia. We analysed the discourses on adolescents’ sexuality and sexual health building upon Gagnon and Simon’s conceptualization of sexual scripts [4].

### Conceptual framework: sexual scripts and adolescents’ sexuality

Holistic understanding of adolescents’ sexuality is of utmost importance to developing effective SRHR interventions. Far from approaching sexuality as an “innate natural drive”, in this paper we consider it as socially constructed, and strongly shaped by (and shaping) cultural, social, and political norms [5].

Of interest here is Gagnon and Simon’s concept of sexual scripts – cognitive schema that instruct people how to understand and act in sexual situations [4]. The concept challenges essentializing constructions of sexuality to acknowledge that sexuality and sexual practices are culturally embedded and need to be understood in the intersection between intrapersonal, interpersonal, and cultural sexual scripts [4, 8]. Parker (2010) argues that, while the sexual script and discursive theories differ extensively, sexual scripts, especially the cultural dimension, might be complementary in understanding adolescent sexuality [4–6].

The Scripting theory provides an important framework to understanding adolescent’s sexuality [4]. In literature, the media and everyday interactions we can identify recurring cultural scripts on adolescents’ sexuality that shape how (young) people understand and experience sex and SRHR programs. These include, among others: 1) gendered scripts that undermine women’s reproductive choices by constructing men as rational and women as irrational in decision-making, 2) the representation of women as passive sexual objects while multiple partners for men is seen as a norm, and 3) the construction of the male sex drive as natural and unavoidable and the acceptance of men’s use of sex to prove their masculinity [9–11]. These form part of the dominant sexual scripts that shape people’s thinking and practices around sex. For this study, we used ‘normative’ to refer to sexual scripts and discourse that are dominant (not necessarily because they are the most commonly practiced) and represented as ‘the norm’.

Specifically, normative scripts around adolescents’ sexuality often represent sex both as taboo and dangerous. In other scripts, sex is presented as an obligatory practice that all adolescents engage in and can grant them status - such scripts are also strongly gendered [10]. Frequently, adolescents’ sexuality is also represented as immature, irresponsible and dangerous compared to that of adults, and more prone to governing practices and regulations [9–11]. While normative and hegemonic sexual scripts are not the same as practices, they provide the context that adolescents ‘ride on’ (and eventually challenge) when engaging in sex or refraining from it [4]. Different and contradictory scripts can coexist, and individuals may not necessarily conform to existing scripts but may defy them. It is therefore, of significant importance to identify normative and hegemonic sexual scripts and their effects, not only for improving the design of SRHR interventions for adolescents but also as a first step for challenging harmful sexual scripts.

## Methodology

### Study design

In this study, we used participant-driven photo elicitation in combination with both individual and group interviews (PEIs). The use of a combination of diverse approaches when collecting data on adolescents’ sexuality has been advocated for in previous research [12].

Photo elicitation is a qualitative methodology within the scope of participatory research, in which researchers use photographs or other images as stimuli to solicit responses, insights or reactions from participants [13]. The use of participant-driven PEIs was motivated by several factors. Firstly, this approach reduces the power imbalance between researcher and participants, since participants are given the power to define what is important to them. Secondly, PEIs provide the researcher with an opportunity to use photographs as a tool to expand on a question, and at the same time allow participants to use photos to depict unique dimensions of their lives. Thirdly, the use of photos in an interview has been shown to make it easier to achieve rapport and lessen some of the awkwardness experienced during conventional interviews [14, 15]. Combining PEIs with individual interviews provided adolescents with an opportunity to share information and discuss photographs that they may not feel comfortable about sharing openly in a group. As our interest was to collect both individual and group opinions and discourses on sexuality, we also conducted one focus group discussion per school with our participants.

### Context

This study was conducted in four rural communities in the Central province of Zambia. We purposively selected four communities that had participated in the community arm of the research initiative to empower girls, which we describe in detail below. The population size of these communities ranged from approximately 1600 to 2100. The key infrastructure in the four communities included at least one government school, a healthcare facility, a market area and a police post. All the communities were characterized by high levels of unemployment and poverty.

Approximately 53% of the total population of Zambia is under the age of 18 years, with 58% living in rural areas. According to the 2018 Zambia demographic health survey (ZDHS), the percentage of adolescents girls who have begun childbearing rises from 6% among those aged 15 years to 53% among those aged 19 years; one in seven female (15%) adolescents aged 15–19 years are married (or in a union) compared to only 1% of their male counterparts; 21.5% of married girls aged 15–19 have an unmet need for family planning; HIV prevalence among adolescents and young people aged 15–24 years was estimated at 3.8% (5.6% among girls and young women vs. 1.8% among boys and young men) [16]. Apart from HIV, all the above SRHR challenges were more prominent in rural than in urban areas. In rural Zambia, practices such as early marriage and childbearing are still prominent and very likely to limit girls’ education while favouring boys’ education [1, 16, 17].

In 2014, Zambia adopted a framework of comprehensive sexuality education (CSE) and introduced it to adolescents aged 10 to 19 years attending school in Grades 5–12. Although several studies have shown that CSE can contribute to overcoming SRHR information barriers, its implementation in Zambia is affected by cultural norms, dominant discourses on adolescent sexuality and lack of clarity about how to integrate comprehensive sexuality education within general school subjects [18]. In Zambia, same sex sexual practices are criminalized, and existing plans related to SRHR, including the CSE curriculum, are limited to heterosexual sexuality.

This study draws on data collected during a larger project that explored how community-based interventions for strengthening adolescents’ SRHR can be integrated, sustained, and made responsive to adolescents within a community-based health system [19, 20]. The broader study was nested within the full intervention arm of the Research Initiative to Support the Empowerment of Girls (RISE) which aimed to support girls to continue attending school, and increase girls’ opportunities to postpone pregnancy and marriage. Details of the RISE intervention have been described elsewhere [20, 21], but in short, in 2016, RISE project recruited girls in grade 7 (regardless of age) and provided them with SRHR information for two (2) years. The project used teachers and community health workers to deliver ‘comprehensive’ SRHR education to adolescents. This SRHR education focused on increasing life skills and knowledge on SRHR including promoting a change in behaviour and beliefs relating to contraceptive use among school and non-school going adolescents. In addition, RISE also held community dialogue meetings with parents aimed at supporting positive SRHR adolescents’ norms coupled with economic support to both the girls and their families [21].

### Participants

**Fig. 1:**
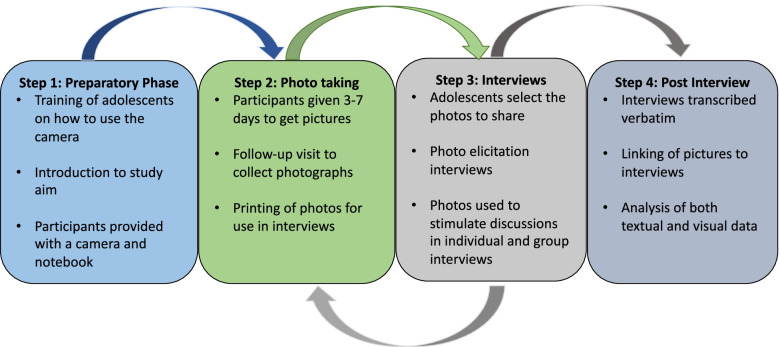
Summary of the PEI data collection procedure as adopted in this study

Twenty-five school-going adolescents aged 16 to 18 years who were attending year 9 (the last year of basic education) were recruited from four schools to participate in the PEIs (Fig. 1), in-depth interviews and focus group discussions (Table 1). The first author (CM) contacted the teachers who at the time were facilitating the RISE comprehensive sexuality education curriculum to help identify adolescents who were participating in the schools’ youth clubs. From the list of potential participants, the researcher then purposively selected six participants per school, making sure to include both boys and girls, from diverse socioeconomic backgrounds and with diverse self-reported experiences in terms of accessing SRHR services in the community. Our final list of participants consisted of six adolescents each from communities 1, 2 and 4 and seven from community 3.

**Table 1:** Number of participants per school

School	Participants (Aged 16–18 years)			IDIs^a^	FGDs^b^
	Female	Male	Total		
School 1	3	3	6	6	1
School 2	3	3	6	6	1
School 3	4	3	6	6	1
School 4	3	3	7	7	1
Total	13	12	25	25	4

^a^In-depth interviews; ^b^Focus group discussion with 6 participants in schools 1, 2 and 3 and 7 participants in school 4

### Procedure

Data collection took place between August and November 2018. We adopted the four stages of PEIs (preparation, taking the photographs, the interview and post interview) as described by Overmars-Marx et al., [22] and adjusted them to suit our study (Fig. 1). The first author (CM) facilitated stages 1–3 of the data-collection process with the help of a trained research assistant. Stage 4 was conducted by the first author together with all the co-authors.

Whereas we do not claim that our study was fully participatory, we took many strategies to engage the participants and alleviate power imbalance. First, we held the one-day training workshop with participants. Although the overall theme of the study was predefined by the authors based on data obtained from the RISE project, the feedback obtained during the workshop enabled us to re-shape both the theme and the interview guide. The first part of the workshop was focused on building acquaintanceship in the hope of making adolescents comfortable and alleviating the power differential between researchers (adults, professionals, coming from the city) and participants (younger, students, rural). Second, we equipped participants with cameras and asked them to take pictures of any part of their lives that could help them to illustrate how they construct, understand and view sexuality and sexual health. We encouraged participants to use pictures of objects and living things that would express their views. Third, during in-depth interviews, we used pictures to start the discussions around sexuality. Participants were invited to select the photographs they wanted to discuss, described what the photograph represented, why they had taken it and how they wanted to apply the photo in relation to the study. The interview guide containing open-ended questions with thematic areas previously discussed during the workshop, was used to elicit more information. In-depth interviews were conducted after the researchers had had several interactions with adolescents during the photo taking period which lasted approximately 6 weeks. Interviews were held in a private space within the school area but out of reach from the teachers or community health workers. Each interview lasted approximately 30 to 45 min.

The interviews were conducted in a mixture of English – which is an official language spoken in schools – and Bemba, a local language spoken by all the participants and a mother tongue for the first author. This made participants more comfortable during the interviews. Furthermore, during the workshop, we provided the study objectives and open-ended interview guide to the participants. Based on the questions discussed during the workshop, some participants were able to lead the discussion. The interviewer supported such participant by probing further based on the information the participant provided.

We ended our data collection by bringing together all the participants in each school for a group discussion. Similar to in-depth interviews, photos were used to begin the discussion. The group discussions lasted approximately 60 to 80 min. We conducted a total of four group discussions (one per school). During data collection, no titles or real names were used during the discussions – all participants including the researchers were called by pseudonym. All the interviews were tape recorded and later transcribed verbatim. The interviews were then linked to the pictures by the first author (CM).

### Data analysis

In this study, the aim of the analysis was to capture both textual and visual data. We adopted the analytical approach of interpretative repertoires, a stream of discourse analysis which is rooted in discursive psychology [23]. Discourse analysis focuses on the construction of identities, how identities arise and how they are negotiated. Discourse analysis treats language not as a mean to communicate ‘truth’ practices or experiences, but as the ‘building blocks’ that people use to make sense of their experiences; language is not reflecting the truth but constructing some ‘truths’ as possible. Interpretative repertoires specifically, have been described as ‘clusters of meanings that people utilize to build up their arguments in a way that makes sense for them’ (pg. 90) [24]. Since repertoires are (re)constructed within moment-to-moment interactions, they are characterized by variability, inconsistences, and contradictions [23, 25]. Thus, the analysis does not focus on determining whether an interpretative repertoire is “true” or “false” but on analysing practices through which repertoires are constructed to appear.

In this study, we focused on analysing constructions of adolescents’ sexuality and sexual health and the consequences of these discourses for their exercising of their SRHR. Understanding discourses on adolescent’s sexuality is important, not because they reflect ‘reality’, perceptions or lived experiences but because they form the ‘foundation’ through which adolescents make sense of their sexuality. Consequently, discourses, influence how adolescents live and make decision in relation to sexuality. By using discourse analysis and scripting theory, we aimed to bring forth existing discourses that influence how adolescents construct their sexuality. Although combining discourse analysis and scripting theory could be regarded as conflicting, we agree with Jackson, who argues that these two approaches could be complimentary [5].

Data analysis started alongside data collection, that was conducted by the first author (CM) with periodic consultation with the co-authors (JMZ, AHK and IG). The first author (CM) together with the co-authors AKH and IG, read the first interviews from the first school and then met to discuss initial codes. Then, the first author continued coding all the interviews from the other schools while reading and re-reading transcripts. The aim of the coding process was to identify statements about sexuality and the consequences of these statements for adolescents’ SRHR. Then, initial codes were periodically discussed among all the co-authors (CM, JMZ, AKH, and IG) to identify interpretative repertoires, focusing more on meanings in the adolescents’ statements, how they expressed themselves and how they built their arguments. We used the technique of identifying “crisis points” and “pronouns” (i.e., signs of conflict, repeating statements and use of words such as “I”, “we”, “they”). The preliminary repertoires were then discussed among all co-authors to identify dominant and contradictory statements. The second step involved going through the pictures that had been selected and interpreted by the participants to identify pictures that related to each of the identified preliminary repertoires. Through regular discussions, we arrived at the final repertoires presented in this study.

## Results

We developed three interpretative repertoires: sex is for mature people; gendered (dis) respectful behaviour; and acquiring and using knowledge on sexuality. These repertoires are not an untouched account of what young people think or their actual practices, rather they mirror normative discourses around adolescent’s sexuality in Zambia, that have potential effects on young people’s sexual practices.

### Sex is for mature people

This repertoire represents adolescents as young and “immature individuals”, not qualified to engage in any form of sexual activity. This construction of “immaturity” was mainly based on age (being young) and being in school. Being ‘immature’ was portrayed as a subject position in which one needed to avoid discussing certain topics about sexuality, and abstain from sexual activity, since one was not old enough to make informed or correct decisions. As a consequence, school-going adolescents who became pregnant or got married were positioned as immature people who had made “bad” decisions. Whereas what was not available/appropriate for “immature” positions appeared to be clear-cut, the exact age or conditions when one could emerge from the position of immaturity was less clear, although references to the age after one completes school and gets married appeared during interviews.

**Fig. 2:**
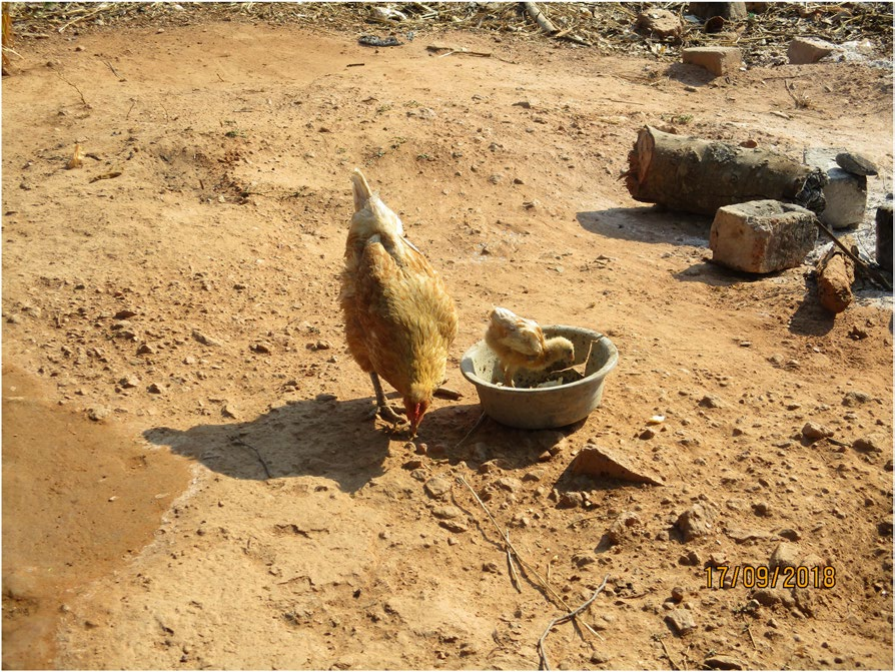
A hen and chick feed in the yard

In the pictures, adolescents depicted themselves as a young chick, young plant or tree and stressed that they were still growing and thus not mature enough to make “good” choices about their own sexuality. This was done while constantly comparing adolescents against their parents or guardians, whom they considered to be mature. Twinkles described this using the photograph he had captured of a hen and a chick (Fig. 2):

*…….I put the picture of the mother (chicken) showing the baby (chick) how to eat…this chicken was looking out for its children (chick),….even us, we are young and not mature, our parents look after our sexual health…our parents teach us ‘this is bad, this is good’. This is how they live, this is how they behave…… So, one of the positives that parents show us… is how to look after ourselves in terms of sexual and reproductive health.* (IDI, Male, School 1)

**Fig. 3:**
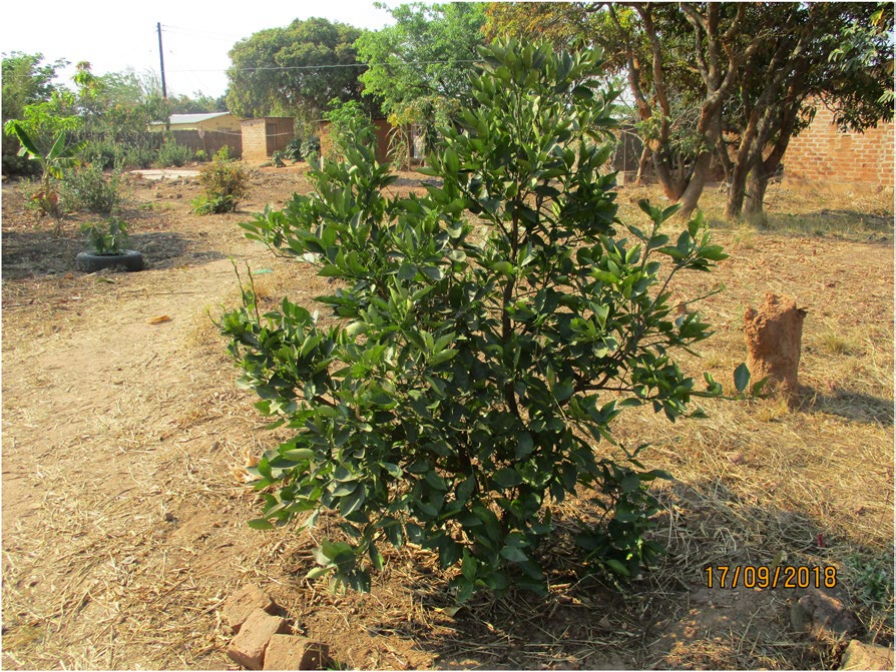
Sate’s photograph of a young plant

However, maturity was also something that adolescents could acquire in ‘appropriate spaces’. Contradicting the statements that certain topics on sexuality can only be discussed when people are “mature”, discussing sexuality in the youth clubs at school was constructed as a maturing and empowering experience. However, notice the focus on the importance of ‘appropriateness’; conversely information and discussion in spaces deemed as ‘inappropriate’ (e.g., without adult supervision and control) may not confer maturity. When asked what this meant, Sate explained using this photograph of a growing tree (Fig. 3):

*I can say we have grown now, because of this youth club that has come. Actually, we are maturing in terms of gaining knowledge, just like this young plant [referring to the picture].* (IDI, Male, School 3)

The maturity acquired from school youth clubs was limited to acquiring knowledge about (certain aspects of) sexuality. The SRHR information that was discussed in the youth clubs, such as condoms and contraceptive use, was represented as information that was meant for use, not at the present time, but in the future. The focus was put on “not having sex” and promoting abstinence for adolescents, as the ways for “not becoming pregnant” as the words of Felemeza portray:

*When we were attending the youth clubs …… they were teaching us [about] contraceptives and condoms, but they did not mean that we should be practicing. They said that that knowledge is for us to use in our future time. Maybe when we complete our education, when we are at the right age to get married... not now.* (FGD, School 4)

**Fig. 4:**
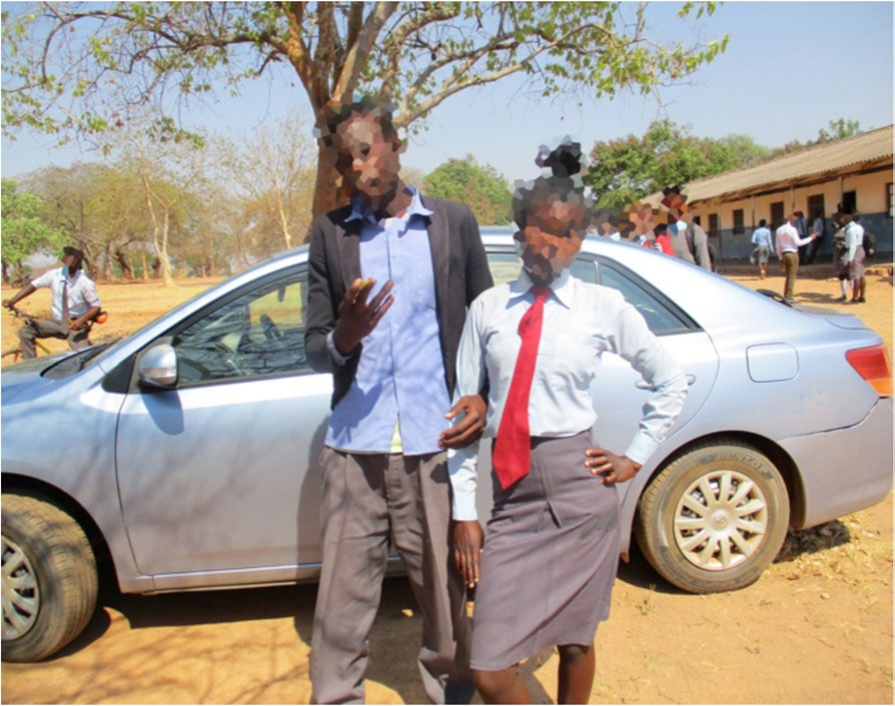
Male and female students linking their arms

This repertoire represents that for adolescents, engaging in sexual activity was “immature” and consequently dangerous and a “highway” to negative consequences, where the risk of getting pregnant (for girls) overshadowed other potential problems. For example, Felemeza showed a staged picture (Fig. 4) she had taken of a boy and girl with their arms linked as they would do if they were in a romantic relationship. Reflecting on the picture, Fros B commented:

*…. the girl when she is pregnant and stops going to school, so she starts suffering with the pregnancy until she delivers, after that she becomes sick with an incurable disease… they are more scared of getting pregnant…* (IDI, Female, School 3)

**Fig. 5:**
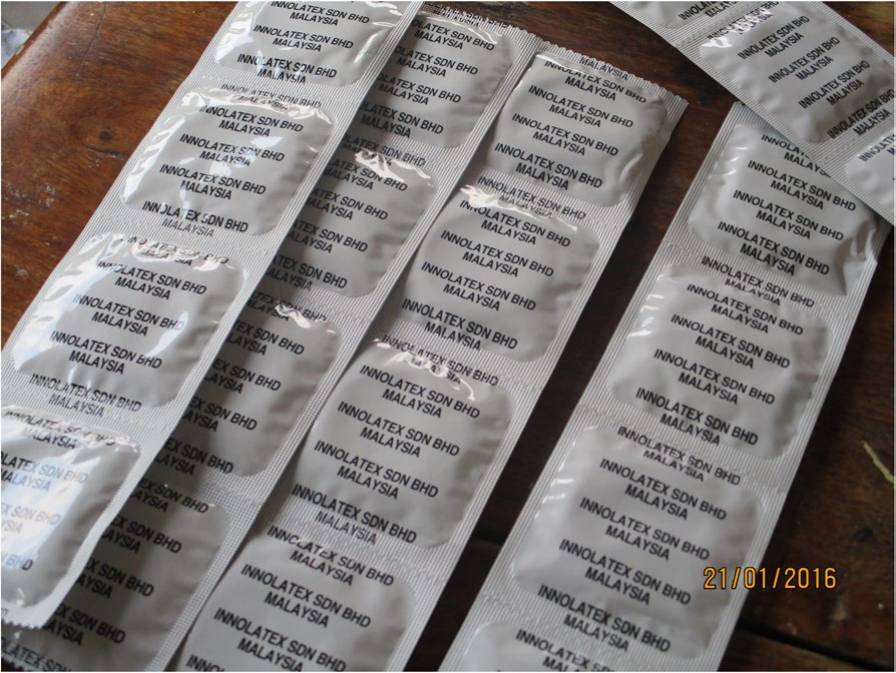
Picture of male condoms

As a consequence of this representation of adolescents as immature, seeking certain SRHR services such as contraceptives and condoms was constructed as an action that can be taken only when adolescents reach an acceptable age (after completing school), while seeking information related to abstinence was portrayed as “normal” and “acceptable”. In one of the FGDs, a photograph of condoms taken by Charlie (Fig. 5) triggered discussions around what SRHR services adolescents can ask for if they visit a health facility. During the discussion, Charlie explained some of the issues an adolescent had to consider before requesting condoms and contraceptives at the health facility as follows:

*You just have to check your age. When you go there [to the health facility], you don’t have to ask the doctor, the nurse about things like condoms, contraceptives because she will tell you bad things [meaning shout at you] and you are going to be upset. You just have to go there to seek [SRH] information according to your age. You should check your age before you…ask for condoms and contraceptives.* (IDI, Male, School 3)

Using the discourse “Sex is for mature people”, adolescents ‘learn’ and internalize norms of what is socially appropriate to ask for and what is not and may expose them to embarrassment or shame. Teresa and Franko elaborated further on this point, in relation to the consequences that it brought for them in terms of how they might be approached by healthcare professionals if they went against this discourse:

*It is not good at our age, you can even feel embarrassed doing it, or going to the hospital asking for condoms as young as I am. How can the doctor feel…looking at me asking for a condom? It is not good.* (IDI, Female, School 4)

### Gendered (dis)respectful behaviour

**Fig. 6:**
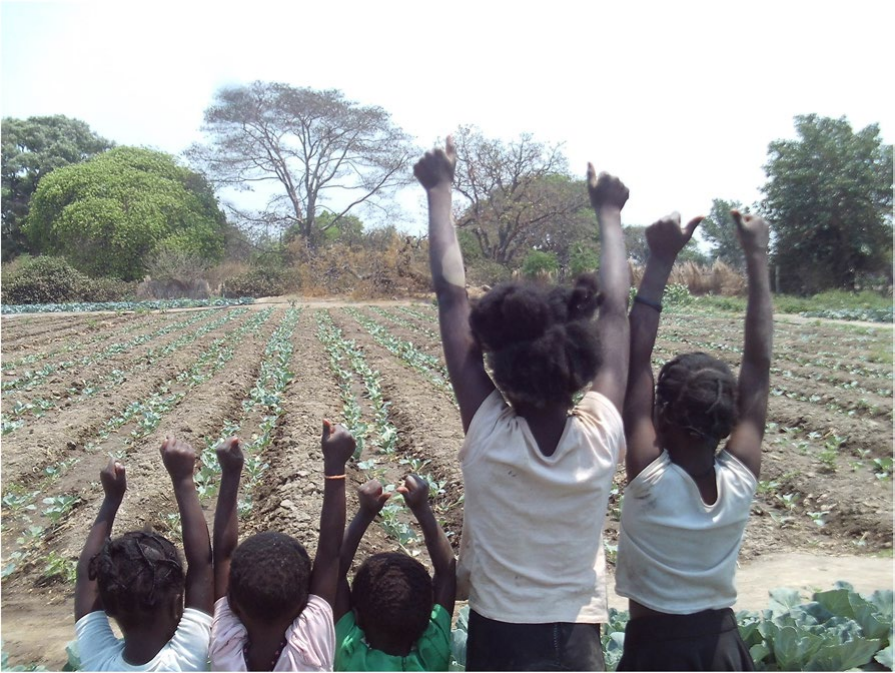
Children displaying the symbol of the slogan “Abstinence is great!”

Sex during adolescence, besides being represented as dangerous and a signal of immaturity, was also constructed as a way of being disrespectful towards parents/guardians. The construction of what was considered (dis)respectful behaviour in relation to sexuality was strongly influenced by gender, and more clearly defined for girls than it was for boys. For girls, respectful behaviour was defined as not engaging in sexual intercourse, not getting pregnant, dressing “appropriately” and staying at home when not at school. To occupy the position of a “respectful girl” demanded fulfilling all of these standards, and there were consequences attached to diverging from them. Respectful girls were labelled as “good” girls, while those who did not conform to these standards were “bad” girls. To illustrate this, Cath showed a photo (Fig. 6) she had taken of children displaying the symbol she described as “abstinence ili che” (meaning abstinence is great). When asked why she had taken that photo, she responded with passion:

*Good girls? They can’t have sex because they are still young, and they are still living at their parents’ house. I think it’s not the right thing to do. It’s not respecting their parents* (IDI, Female, School 3)

**Fig. 7:**
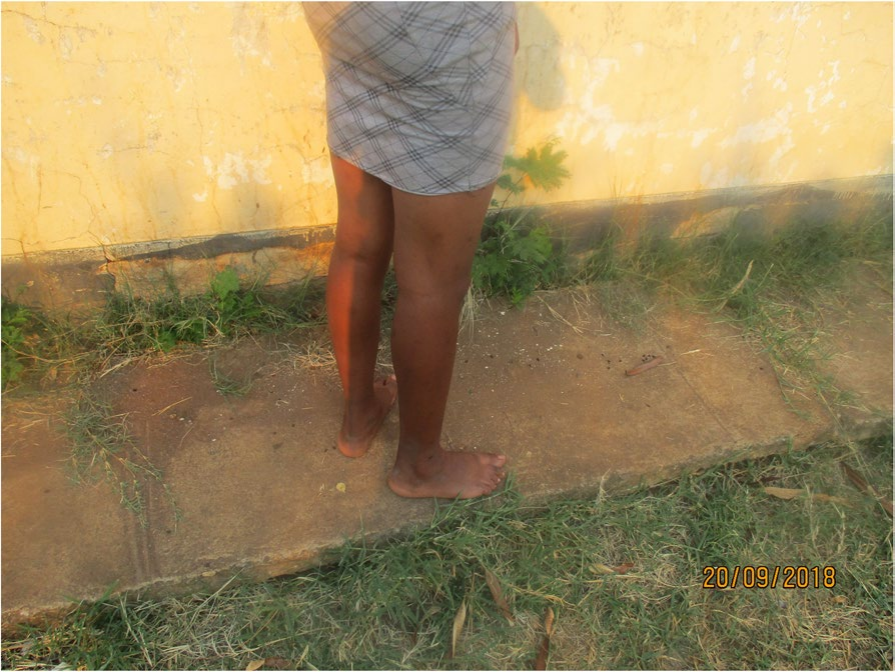
Girl wearing a mini skirt

Photographs also emphasized the importance of clothes and body presentation as “markers” of respectability for girls and women. Felemeza, for example, took and chose a picture of a girl wearing a miniskirt (Fig. 7) as a symbol of being a “bad girl” and elaborated:

*She’s [pointing at the picture] the girl in the miniskirt….If you sleep with men, you will become like her wearing miniskirts looking for boys…You will stop obeying adults like her. She has lost respect …. She is not a good girl.* (IDI, Female, School 2)

Very few statements were made in reference to disrespectful (or respectful) behaviour for boys. Where this was done, respectful behaviour was defined mainly as studying and getting good grades at school. As expressed by Twinkles:

*…. but my mum she doesn’t say anything...…… Actually, all she talks to me about sex is how to be a good boy, like how to behave and how to be smart in school… My dad doesn’t say anything.* (IDI, Male, School 1)

Boys were portrayed as victims of the “bad” girls, while “bad” girls were constructed as “seducers” of the boys. At the same time, heavy expectations were placed on adolescent girls to protect themselves from the negative consequences of sex and pregnancy. For example, it was expected that girls and not boys should abstain from sexual activity, dress “appropriately” and stay away from places where boys and men are found. As girls could get pregnant and boys could not, the responsibility for avoiding the consequences of sex were seen as a girl’s duty. As McZee explained:

*So, when you have sex with a girl, she will fall pregnant. A boy does not become pregnant, so you won’t fall pregnant. When you make that girl pregnant you will make her suffer.* (IDI, Male, School 4)

### Acquiring and using knowledge about sexuality

In this repertoire, having knowledge about sexuality was portrayed as essential for preventing most of the unwanted pregnancies and early marriages during adolescence. Our participants positioned girls and boys who accepted and practiced abstinence as having knowledge while those who engaged in any form of sexual activity as lacking knowledge. Engaging in sexual activity was constructed as a consequence of lacking knowledge, which could result in unwanted pregnancies and diseases. In the same way that the conditions for emerging from immaturity was not clearly defined, the specific knowledge needed for one to be considered as knowledgeable or lacking in knowledge was not clearly defined too.

Pregnant and sexually active girls were thus portrayed not only as immature and disrespectful, but also as girls who did not pay attention to what was taught, and who lacked understanding and knowledge. Mira’s photograph offers a good example of how images were used to illustrate the position of lacking knowledge and its consequences. Mira took a photo of her friend (who had consented to be photographed) holding her baby of 4 months to illustrate how lack of knowledge can lead to adolescent pregnancies. Commenting on Mira’s picture, Skyfresh reflected:

*… You know, sometimes those people who are pregnant – it’s not their fault. It’s actually lack understanding … that when you have sex without a condom you can get pregnant* (IDI, Female, School 2)

However, there were nuances to the representation of unplanned pregnancies caused only by lack of knowledge. In the group discussion, the position of lacking (or having enough) knowledge and how it can influence uptake of SRH services (for sexually active adolescents) was rigorously debated. Some participants argued that being pregnant or sexually active was not always a consequence of lack of knowledge. One of the key determinants of adolescents’ pregnancies identified in all the four communities was poverty. Poverty was portrayed as a factor that can contribute to adolescents engaging in unsafe sex so as to earn money for their basic needs.

*“Like the other girls they fall in love (and engage in sex) because they lack things. You find that a girl is (poor)…always having trouble…. the parents have died, maybe 15 years ago…. the stepmother is mistreating the girl, so the girl will use the money to buy basic needs” (FGD, School 2)*

In addition, participants highlighted that, even with knowledge, gaining access to “certain kinds of” sexual and reproductive health services, such as contraceptives and condoms, was still challenging. The fear of being stigmatized by others when accessing certain types of SRHR services such as contraceptives, and the perception of the health workers as unfriendly and un-confidential, exacerbated the lack of access to condoms and contraceptives and consequently resulted in adolescent pregnancies. As Lee questioned:

*“…. uhm (thinking)… … I don’t know but most of the girls are afraid to go to the clinic. They can shout at you “what are you doing here at your age” …. You are supported to be in school, why do you want family planning (meaning contraceptive)?”. (FGD, School 3)*

## Discussion

The repertoires we found represent adolescents and adolescent’s sexuality in quite a negative and deficitary way. Adolescence was represented as a period when people are not old enough to make informed decisions about their own sexuality, they should avoid discussing certain topics relating to sexuality and need to abstain from sexual activity. The fact that such repertoires emerged from discussions with adolescent participants can be interpreted as a sign of how powerful normative discourses have become. Our findings paint quite a bleak picture but are in no way unexpected; as Cuervo and Wyn argue, adulcentric discourses in which adolescence is conceptualized as a transitional and “risky” period in life during which young people progress from one status to another, remain normative [26]. In our study, sexuality was portrayed as not only a “risky” area for adolescents to navigate through as they transition to adulthood, but also as a space for respectability for girls and young women.

The three repertoires developed from the interviews with young people have to be understood as building upon larger normative and hegemonic discourses holding on to moral standards of sexual abstinence that most likely do not align with young people’s everyday experiences of engaging in sex voluntarily or through coercion or violence. From our analysis, we did not find any strong resistance/challenging discourse, but rather that all the repertoires aligned with Christian fundamentalism which equally expect and uphold abstinence as the ‘gold standard’ for unmarried adolescents [21, 27]. Such discourses have contributed to adolescents shunning away from accessing the much-needed information and services [1]. Despite such hegemonic discourses, it is important to highlight that some of the participants insisted on the importance of learning and obtaining comprehensive information and access to services as a right for all adolescents.

Similar to other studies, adolescents’ talk about sexuality was mainly constructed within public health discourses that focus on ‘risks related to engaging in sex’, such as early pregnancy, and sexually transmitted infections [28, 29], and leave other key aspects of sexuality silenced. Although previous studies have highlighted how sex for pleasure, fun or love is intrinsically connected to sexual experience, very little discussion on such appeared in our material. It is possible that our participants did not feel comfortable to freely express their thoughts on sex for pleasure, fun and love because of the perceived need to conform to the sexual scripts that promote abstinence and delayed sexual debut for adolescents. The need to conform to normative sexual scripts could hinder adolescents from engaging in meaningful conversations around sex that go beyond abstinence promotion and risk prevention. It could also prevent adolescents from accessing SRHR information and services that could help them engage in safer sex. As Clief (2020) argues: ‘unless sexuality education is positioned in the broader scope of sexual experience, it is unlikely to fully and effectively engage young people’ (pg. 8) [30].

Constructing sexuality in terms of age and maturity has serious implications for how adolescents adopt SRHR interventions. An emphasis on age and maturity, coupled with teaching adolescents about sexual risks and the dangerous outcomes of sex, could open up the possibility of delayed sexual debut due to fear, but at the same time it stigmatizes adolescents’ sexual practices and discourages them from seeking SRHR services [2, 31]. Defining certain forms of information as inappropriate for adolescents also reinforces the notion that young people are not autonomous, and consequently limits their SRHR choices.

Our repertoires also display a gap between adolescents’ having knowledge and applying it. The solution to preventing pregnancy for sexually active adolescents was represented as acquiring “more” SRHR knowledge and listening to advice from parents, despite the fact that adolescents still considered discussing certain types of SRH topics with their parents’ ‘taboo’ due to their “immature” situation. In this study, “having knowledge” was equated to abstinence and “not having” sex in any form before marriage.

Although adolescents talked about sex for compensation and early marriages as resulting from poverty, the ‘cause’ for adolescent pregnancies was still portrayed as mainly one of lacking knowledge. This focus on ‘imparting (partial) knowledge’, as ‘the solution’, is problematic. Individuals’ autonomy and ability to exercise their SRHR cannot be ensured by imparting knowledge to adolescents alone. It must be achieved through using a combination of services, counselling and information that respond to their needs in a friendly way, and these should be implemented in a way that recognizes the immediate, distal, social and cultural factors that shape adolescents’ values and behaviour [2, 7].

Our study also found that sexual scripts are strongly gendered and disadvantage girls and young women. Female adolescents were portrayed as “bad” or disrespectful to adults when they engaged in sex, while male adolescents were portrayed as victims of these “bad” or disrespectful girls. This finding was unexpected because most studies that have explored heterosexual sexuality portray men as dangerous and women as victims in need of protection [11]. Similar to other studies, we found that the responsibility for protecting oneself from pregnancy and sexually transmitted infections was principally placed on girls, ignoring contextual factors and gendered power relations at play within the setting [32]. These gendered sexual scripts constrain young women’s (sexual) agency and have been shown to affect the way in which comprehensive sexuality education (CSE) is implemented in Zambia and neighbouring countries.

Recent evaluations of other CSE programs indicate that these interventions are commonly characterized by approaches that highlight the dangers, risks, and negative consequences of sex, in which the responsibility is placed on girls’ shoulders [9, 18]. In this study we do not report how much RISE was implemented. Thus, the evaluation on the implementation of RISE and whether teachers and CHWs adhered to the “progressive” planned approaches of implementing sexual education as highlighted in the RISE protocol is beyond the scope of this study [21]. However, a similar study on CSE (but not linked to RISE) showed that some teachers used discretion to decide what and when to teach CSE which resulted in holding back certain information from adolescents [18].

We found that when sexual activity resulted in negative outcomes (i.e., unwanted pregnancies, early marriages or sexually transmitted infections), the blaming fall on the girls, their dressing and actions while boys/men walk free without any questioning. Such double standards that harm and limit girl’s autonomy have been pointed out repeatedly in the literature [8, 10, 11, 17, 28]. More research is required to understand how these inequities in expectations of men vs. women influence how messages are perceived, especially in SRHR interventions that target adolescents and aim to reach both males and females equally. We agree with Sheffer and Foster [9], who argued that the mere act of adding sexuality education to the school curriculum does not guarantee a constructive or appropriate approach to addressing adolescents’ knowledge gaps because many teachers tend to re-inscribe the negative sexual scripts that perpetuate gender inequalities, stereotypes, and stigma into their messages [9].

Finally, we found that some aspects of sexuality, such as non-heterosexual sex, were silenced in the discussions with our participants. As described in previous studies [33, 34], this could be attributed to hegemonic heteronormative discourses that represent heterosex as “the norm” and ‘the normal’. Heteronormative discourses produce (and are reproduced by) policies, laws and regulations intertwined with Christian fundamental, such as the law that criminalizes homosexual sexual relationships in Zambia. There is a need for future studies to explore the extent to which heteronormativity limits SRHR and access to services for LGBTQI+ adolescents.

### Implications for SRHR interventions

The first step to challenging normative and hegemonic discourse is to make them visible. By analysing discourses on adolescents’ sexuality and sexual health and the consequences of these discourses for adolescents’ exercising of their sexual and reproductive rights, we contribute to visibilizing normative discourses to program implementers, and other stakeholders targeting adolescents SRH in Zambia and other similar settings. We think visibilizing normative discourses and their ‘effects’ on sexuality, can start the process of challenging them. As this study has shown, three normative discourses influence how adolescents in Zambia and similar settings negotiate, adopt and/or resist SRHR interventions.

First, the effects of constructing sexuality in terms of age and maturity have serious implications on how comprehensive sexuality education and other SRHR interventions are implemented in Zambia. Effective interventions that aim to promote adolescents’ SRHR should be comprehensive, instead of being limited to promoting abstinence as the only acceptable knowledge, even though this might conflict with dominant sexual scripts. Such interventions should be sensitively tailored to address the needs of diverse sub-groups of the adolescent population, and critical with adulcentric perspectives that depict young people as immature and irresponsible.

Second, sexual scripts are influenced by gender and inequitably differentiate what is considered ‘appropriate’ for boys and for girls. This means that there is an urgent need to include into comprehensive sexuality education discussions about gender issues, about consent, about normative gendered expectations, about masculinity/femininity, and rights. Such discussions must stress that everyone bears a responsibility when engaging in sex (and reproductive decisions) and everyone has a right to have their sexual and reproductive rights fulfilled, and that requires to tackle unequal gender expectations and norms. Challenging normative gendered sexual scripts is a shared responsibility, not only for adolescents but all key stakeholders involved in planning, advocating for, or delivering adolescents SRHR education and interventions.

Lastly, ending teenage pregnancies, early marriages and risky sexual practices cannot be achieved by providing knowledge alone (even comprehensive sexuality education). As this study shows, there are many other structural and symbolic factors that influence how adolescents negotiate, adopt and/or resist SRHR interventions, and how they engage, enjoy, or are forced into sex. Interventions should reflect and plan strategies to tackle poverty, and to ensure access to sexual and reproductive services, to name just a few crucial issues. It is therefore important to ensure holistic approaches are use which address both the social and economic needs, and at the same time ensuring access to knowledge and adolescents’ responsive SRH services.

### Strengths and limitations

Our study used a robust methodology to understand the social construction of adolescents’ sexuality and provides insights that will be useful for intervention strategies, in terms of taking into consideration the pivotal role of cultural scripts in shaping adolescents’ sexuality [11]. This is one of very few studies to combine PEI and discourse analysis to explore adolescents’ sexuality. As described by Shefer and Foster, [9] the use of PEI enhances qualitative inquiry, while discourse analysis allows for a detailed analysis.

This study was not without challenges. Although most adolescents found the use of cameras and photo elicitation acceptable and even exciting, a few acknowledged that the process was time-consuming and felt stressed regarding what pictures to take. This was consistent with the findings of other authors who have used PEI [14, 15]. To alleviate this discomfort, guidance was provided, and participants were encouraged to take pictures at a time when they felt comfortable. Because of the potential to include individuals who were not willing to be photographed, adolescents were asked to limit identifying features when taking photos. This was experienced as limiting by some participants, who wanted to share photographs that included friends and family. In instances where participants ignored this and brought pictures that included other people, we obtained informed consent and assent (if needed) from those persons. All pictures that were submitted with identifiable features were de-identified by the researcher.

A limitation that we acknowledge is that the researcher could have been considered as an outsider from the city in the rural schools. Although the researcher was not part of the RISE implementing team, adolescents could have perceived her as being part if the RISE team. This could have made power imbalance between researcher and participants larger than what is desirable in PEIs. Such perceptions might have also resulted in participants avoiding uttering resisting or challenging discourses on sexuality, and/or being critical towards the RISE intervention. In addition, participants could have felt the need for social desirability - that is, offer answers as they had been taught in the RISE intervention. To try and mitigate this, the researcher (CM) explained to the participants that she had no relationships with the RISE implementation team and re-assured adolescents of their confidentiality.

Finally, through depicting normative discourses on adolescent sexuality, we may have contributed to reinforce them. However, as mentioned before, we consider that visibilization of normative sexual scripts about sexuality is the first and necessary step for challenging them.

## Conclusion

This article reveals how adolescents construct their sexualities and sexual health, and the consequences of these discourses for adolescents’ exercising of their sexual and reproductive rights in rural Zambia. We found that adolescents portrayed themselves as “immature”, not old enough to engage in sex or make decisions about their own sexuality. Constructions of sexuality were influenced by gender and were more clearly defined for girls than for boys. Consequently, the adoption of SRHR knowledge and decisions to access services were influenced by sexual scripts around adolescents’ sexuality, including how and what information and services were considered available/appropriate for adolescents. Future interventions that target adolescents’ SRHR must aim to address the sexual scripts that have serve to erect barriers against positive sexual behaviours, including access to SRHR services that promote safer sex.

## Data Availability

The data for this study can be accessed by sending a reasonable request to the corresponding author.

## Abbreviations

CSE: Comprehensive sexuality education
ERES: Excellence in research ethics and science
FGD: Focus group discussion
IDI: In-depth interviews
PEIs: Photo elicitation interviews
RISE: Research initiative to support the empowerment of girls
SRHR: Sexual reproductive health and rights
ZDSH: Zambia demographic health survey
